# Oral ibandronate reduces the risk of skeletal complications in breast cancer patients with metastatic bone disease: results from two randomised, placebo-controlled phase III studies

**DOI:** 10.1038/sj.bjc.6601663

**Published:** 2004-02-24

**Authors:** J J Body, I J Diel, M Lichinitzer, A Lazarev, M Pecherstorfer, R Bell, D Tripathy, B Bergstrom

**Affiliations:** 1 Institut Jules Bordet, Université Libre de Bruxelles, Brussels, Belgium; 2 CGG-Klinik GmbH, Mannheim, Germany; 3 Cancer Research Center, Moscow, Russia; 4 Oncological Center, Barnul, Russia; 5 Wilhelminenspital, Vienna, Austria; 6 The Andrew Love Cancer Centre, Victoria, Australia; 7 University of Texas Southwestern Medical Center, Dallas, TX, USA; 8 Hoffmann-La Roche Inc., NJ, USA

**Keywords:** ibandronate, oral bisphosphonate, bone metastases, breast cancer, skeletal complications

## Abstract

Although intravenous (i.v.) bisphosphonates are the standard of care for metastatic bone disease, they are less than ideal for many patients due to infusion-related adverse events (AEs), an increased risk of renal toxicity and the inconvenience of regular hospital visits. The use of oral bisphosphonate therapy is limited by concerns over efficacy and gastrointestinal (GI) side effects. There remains a clinical need for an oral bisphosphonate that offers equivalent efficacy to i.v. bisphosphonates, good tolerability and dosing convenience. Oral ibandronate, a highly potent, third-generation aminobisphosphonate, has been evaluated in phase III clinical trials of patients with bone metastases from breast cancer. In two pooled phase III studies, patients with breast cancer and bone metastases were randomised to receive oral ibandronate 50 mg (*n*=287) or placebo (*n*=277) once daily for up to 96 weeks. The primary end point was the skeletal morbidity period rate (SMPR), defined as the number of 12-week periods with new skeletal complications. Multivariate Poisson's regression analysis was used to assess the relative risk of skeletal-related events in each treatment group during the study period. Oral ibandronate 50 mg significantly reduced the mean SMPR compared with placebo (0.95 *vs* 1.18, *P*=0.004). There was a significant reduction in the mean number of events requiring radiotherapy (0.73 *vs* 0.98, *P*<0.001) and events requiring surgery (0.47 *vs* 0.53, *P*=0.037). Poisson's regression analysis confirmed that oral ibandronate significantly reduced the risk of a skeletal event compared with placebo (hazard ratio 0.62, 95% CI=0.48, 0.79; *P*=0.0001). The incidence of mild treatment-related upper GI AEs was slightly higher in the oral ibandronate 50 mg group compared with placebo, but very few serious *drug-related* AEs were reported. Oral ibandronate 50 mg is an effective, well-tolerated and convenient treatment for the prevention of skeletal complications of metastatic bone disease.

Intravenous (i.v.) bisphosphonates are the standard of care for patients with metastatic bone disease, with proven efficacy in reducing skeletal complications (Hortobagyi *et al*, 1998; Theriault *et al*, 1999; Hillner *et al*, 2000; Lipton *et al*, 2000; Rosen *et al*, 2001; Pavlakis and Stockler, 2002; Body *et al*, 2003c). Yet, for many patients, i.v. bisphosphonate therapy is less than ideal. The risk of infusion-related adverse events (AEs) and the possibility of renal toxicity adds to the treatment burden already faced by patients with advanced cancer. The need for frequent hospital visits and lengthy infusion duration (2 h for pamidronate) can make treatment cumbersome and inconvenient for the patient, particularly as a long-term therapy.

Bisphosphonates administered via the oral route would allow the convenience of self-administration at home. However, oral clodronate has been shown to be less effective than i.v. pamidronate at reducing the risk of skeletal-related events (SREs) (Pavlakis and Stockler, 2002), and its use can be associated with unpleasant gastrointestinal (GI) AEs, especially diarrhoea (Kristensen *et al*, 1999; Powles *et al*, 2002). Owing to its relatively low potency (Green *et al*, 1994), high doses are required and at least two large tablets that are difficult for some patients to swallow have to be taken daily (Paterson *et al*, 1993; Robertson *et al*, 1995). These factors, coupled with a recommended 1-h prefood fasting period, may affect patient adherence to treatment.

Ibandronate, a highly potent, third-generation aminobisphosphonate, has been developed in both i.v. and oral formulations for the management of metastatic bone disease. As reported elsewhere, the i.v. formulation of ibandronate (6 mg infused every 3–4 weeks) has been shown to reduce significantly the risk of skeletal complications, alleviate bone pain and improve quality of life in patients with metastatic breast cancer, in the absence of renal safety concerns (Body *et al*, 2003a, 2003b; Diel *et al*, 2003; Tripathy *et al*, 2003). This paper presents the results of a pooled analysis of two phase III clinical trials that assessed the efficacy and safety of oral ibandronate (50 mg day^−1^) in the treatment of women with breast cancer and bone metastases.

## PATIENTS AND METHODS

### Study population

Two randomised, parallel-group, double-blind, placebo-controlled studies were conducted by centres across Europe and other countries, including Australia and the United States of America. Women with histologically confirmed breast cancer and radiologically confirmed bone metastases were recruited into the studies. Patients were required to have a WHO performance status of 0, 1 or 2, be at least 18 years of age, and to provide written informed consent. The exclusion criteria included previous treatment with bisphosphonates or gallium nitrate within the last 6 months, life expectancy <60 weeks, hypercalcaemia (serum calcium, albumin corrected, ⩾2.7 mmol l^−1^), hypocalcaemia (serum calcium, albumin corrected, ⩽2.0 mmol l^−1^), severely impaired renal function (serum creatinine >3.0 mg dl^−1^), Paget's disease of the bone, primary hyperparathyroidism, known liver/brain metastases, receiving a high-dose chemotherapy (i.e. dose intensity >3 times standard therapy), having a medical history of aspirin-sensitive asthma, or receiving treatment with aminoglycoside antibiotics within 4 weeks prior to the start of study medication.

In the two studies, patients were randomised to treatment with oral ibandronate 20 mg, 50 mg or placebo once daily for up to 96 weeks. Only the ibandronate 50 mg data (*vs* placebo) are reported in this pooled analysis, as 50 mg will be the recommended dose for clinical use. Patients were instructed to take one tablet in the morning 1 h before breakfast with a glass of water, but not with milk, milk products or calcium tablets. To assess compliance with therapy, patients were required to return their oral medication to the investigator every 12 weeks for checking. Concomitant treatments were allowed during the study, except those specified as the exclusion criteria.

### Efficacy and safety assessments

Efficacy and safety data from the two studies were pooled for analysis, as predefined in the study protocols. Fractures, bone pain, analgesic consumption, episodes of radiotherapy and surgical interventions were assessed at 4-weekly clinic visits. Urine samples were collected at weeks 4, 12, 24, 48, 72 and 96 for the assessment of c-telopeptide (CTx), a marker of bone turnover, using Crosslaps™ CTx assay. AEs were recorded continuously throughout the study.

To allow for the possibility of nonrandom withdrawal from study groups, postwithdrawal follow-up (PWFU) efficacy and safety data (i.e. for the period from study withdrawal until death or the last scheduled study visit) were also collected. Postwithdrawal follow-up data collection were discontinued when treatment with another bisphosphonate began.

### Analysis of efficacy

The primary efficacy parameter was the skeletal morbidity period rate (SMPR) defined as the number of 12-week periods with new skeletal complications, divided by the total observation time. Skeletal complications included vertebral fractures, pathological nonvertebral fractures, radiotherapy for bone complications (uncontrolled bone pain or impending fractures) and surgery for bone complications (fractures or impending fractures). To allow for the time spent in the study, SMPR was calculated using a revised event ratio method, as follows (Scott *et al*, 2003):


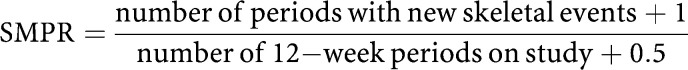


As prespecified in the data analysis plan, analyses of the primary end point excluded data collected in the first 12-week period. Exclusion of early events avoids the loss of power associated with events occurring too early to have been prevented by bisphosphonates. It was anticipated that effects on bone events of ibandronate *vs* placebo would begin to appear 6–8 weeks after drug initiation. The first 12 weeks were selected for exclusion as study visits were on a 3-month basis.

Supportive analyses of the SMPR included the mean number of skeletal events per patient, the mean number of 12-week measurement periods with events per patient, the percentage of patients with skeletal events and time to first new bone event. A multivariate Poisson's regression analysis was performed to assess the risk of developing a skeletal event over the entire 96 weeks of treatment, while controlling for any differences in baseline characteristics between the oral ibandronate 50 mg group and the placebo group. The input variables for the Poisson's regression analysis were country, age, estrogen/progesterone receptor status, performance status, time from breast cancer and metastatic bone disease diagnoses to study initiation, extraosseous metastases, prior hormone and chemotherapy, pathological fractures at baseline, pain score, analgesic score and baseline laboratory measures (e.g. haemoglobin, alkaline phosphatase, aspartate transaminase, white blood cell counts).

### Statistics

The global null hypothesis (no difference in SMPR between ibandronate and placebo) was tested at the two-sided *α*-level of 5% using the nonparametric Jonckheere–Terpstra method (Terpstra, 1952; Jonckheere, 1954). If the global hypothesis was rejected, pairwise comparisons between treatments were performed using the Wilcoxon's rank-sum method, maintaining an overall two-sided *α*-level of 5% and following a closed-test procedure. The trial was designed such that the statistical analysis of the study was powered for the composite end point SMPR, but not for the components of the composite. Efficacy analyses were conducted on the intent-to-treat (ITT) population (all patients randomised) and included PWFU data. Evaluation of safety was based on all randomised patients who had received at least one dose of study drug and had at least one follow-up assessment.

### Ethics

The study was performed in accordance with the principles of the Declaration of Helsinki, the Guidelines on Good Clinical Practice and local medicines legislation in place at the time of study initiation.

## RESULTS

### Patients

A total of 564 patients were randomised to treatment with oral ibandronate 50 mg (*n*=287) or placebo (*n*=277) and were included in the ITT analysis. Patient demographics and baseline characteristics are shown in Table 1

**Table 1:** Patient demographics and baseline characteristics

	**Placebo (*n*=277)**	**Ibandronate, 50 mg (*n*=287)**
*Age (years)*		
Median (range)	56 (26–87)	57 (27–92)

Race, Caucasian (%)	94	93
Median time from diagnosis to first drug intake (years)	3.87	3.44
Median time from bone metastases diagnosis to study entry (years)	0.48	0.46

*Performance status* (%)		
WHO grade 0 or 1	85	84
WHO grade 2	15	16

Mean pain score	1.13	1.33
Mean analgesic score	0.98	1.09
Prior fractures at baseline (%)	43	52

. Although the study groups were generally comparable at baseline, the oral ibandronate 50 mg group contained a higher percentage of patients receiving ongoing cytotoxic therapy, with a higher mean bone pain score and a higher percentage of patients with pre-existing fractures than in the placebo group (differences between groups nonsignificant).

The percentage of patients completing the 96-week treatment period was 42% in the ibandronate group and 38% in the placebo group. The median time on study (from randomisation to study end) was 79 weeks with ibandronate compared with 69 weeks with placebo (NS). The most frequent reasons for withdrawal were malignancy progression (affecting 12% of patients receiving ibandronate *vs* 19% of patients receiving placebo), death (15 *vs* 12%) and other AEs (10 *vs* 12%).

### Efficacy

**Figure 1:**
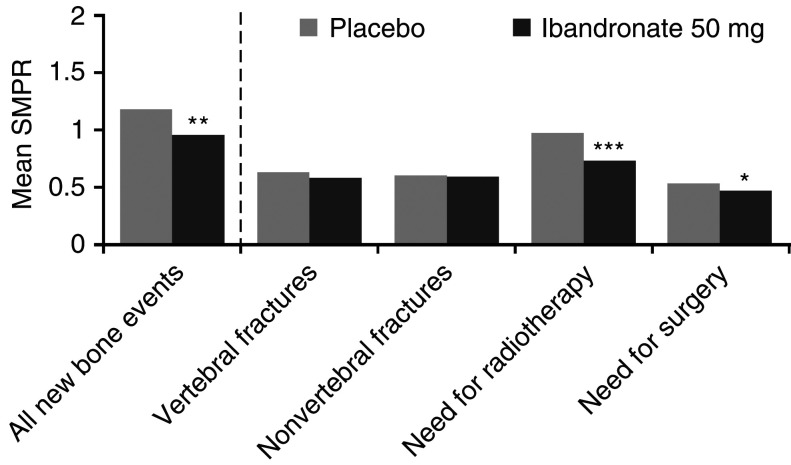
Summary of the mean SMPR, weighted for observation time (^*^P<0.05, ^**^P<0.01 and ^***^P<0.001).

The mean SMPR for all new bone events was significantly reduced with oral ibandronate 50 mg compared with placebo (*P*=0.004) (Figure 1

). Analysis of the individual components revealed that this effect was due primarily to significant reductions in bone events requiring radiotherapy (*P*<0.001) or surgery (*P*=0.037) (Figure 1). There was no significant difference in the number of skeletal fractures with ibandronate compared with placebo (*P*=0.195). When bone events occurring during the first 12-week period were included in the SMPR calculation, the impact of ibandronate on the incidence of skeletal events was reduced, but remained significant for overall SMPR and for events requiring radiotherapy (Table 2

**Table 2:** Supportive analysis of SMPR, including events occurring during the first 12 weeks of treatment

**Mean SMPR**	**Placebo**	**Ibandronate, 50 mg**
All new bone events	1.15	0.99, *P*=0.041*
Vertebral fractures	0.52	0.49, *P*=0.145*
Nonvertebral fractures	0.52	0.51, *P*=0.330*
Need for radiotherapy	0.98	0.80, *P*<0.004*
Need for surgery	0.44	0.40, *P*=0.098*

SMPR=skeletal morbidity period rate.

* Wilcoxon's rank-sum test, pairwise comparisons *vs* placebo.

). Supportive analyses of new bone events demonstrated that the mean number of events and the mean number of measurement periods with events per patient were significantly reduced in the ibandronate group compared with placebo (*P*=0.008 and 0.015, respectively, Table 3

**Table 3:** Supportive analyses of new bone events

	**Placebo**	**Ibandronate, 50 mg**
No. of events per patient	1.85	1.15, *P*=0.008*
No. of 12-week periods with events per patient	0.99	0.71, *P*=0.015*
% of patients with events	52.2	45.3, *P*=0.122*

* Wilcoxon's rank-sum test, pairwise comparisons *vs* placebo.

). The median time to first bone event was 90.3 weeks with oral ibandronate 50 mg and 64.9 weeks with placebo (*P*=0.089).

Multivariate Poisson's regression analysis showed that the risk reduction for a skeletal event in the ibandronate 50 mg group was significantly lower than in the placebo group (hazard ratio 0.62, 95% CI=0.48, 0.79, *P*<0.0001), translating to a 38% risk reduction for ibandronate *vs* placebo.

**Figure 2:**
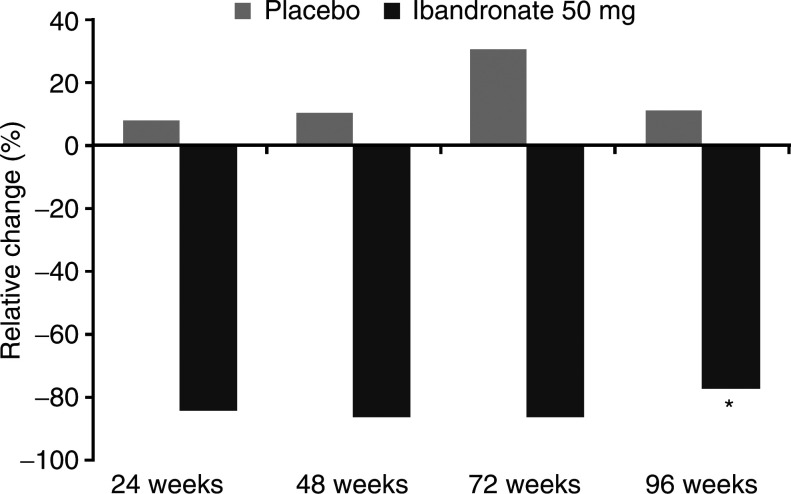
Change in urinary CTx during study period (^*^P<0.001).

Patients receiving oral ibandronate 50 mg showed a significant decrease from baseline in the bone marker urinary CTx over the 96-week study period compared with placebo (median change −77.3% and +11.0%, *P*<0.001) (Figure 2

).

### Safety

A total of 563 patients were included in the safety analysis. As would be expected with skeletal metastases due to advanced cancer, almost all patients reported AEs during the course of the study. The percentage of patients experiencing any AE was similar between the oral ibandronate 50 mg and placebo groups (94.4 *vs* 95.3%). The most frequently recorded AE was malignancy progression (affecting 67.5 and 70.8% of patients, respectively). There was a slightly higher incidence of drug-related AEs with ibandronate (26.6%) than with placebo (17.7%), primarily due to more reports of hypocalcaemia in the ibandronate group (Table 4

**Table 4:** Treatment-related AEs (reported by ⩾2% of patients in any treatment group)

**AEs**	**Placebo (*n* (%))**	**Ibandronate, 50 mg (*n* (%))**
Abdominal pain	2 (0.7%)	6 (2.1%)
Dyspepsia	13 (4.7%)	20 (7.0)
Nausea	4 (1.4%)	10 (3.5%)
Oesophagitis	2 (0.7%)	6 (2.1)
Hypocalcaemia	14 (5.1%)	27 (9.4%)

AEs=adverse events.

), a side effect associated with the use of any bisphosphonate. Serious AEs that were considered to be drug related were experienced by 1.0% of patients receiving ibandronate, compared with 1.4% of patients in the placebo group.

The incidence of mild treatment-related upper GI AEs (dyspepsia, nausea and oesophagitis) was slightly higher in the oral ibandronate 50 mg group compared with placebo (Table 4). The incidence of drug-related upper GI AEs known to be associated with oral bisphosphonate administration was similar in the placebo and 50 mg oral ibandronate groups (Table 4). Only two serious upper GI AEs (duodenal ulcer haemorrhage and nausea, each recorded by one patient) were considered related to ibandronate treatment.

The incidence of renal AEs was comparable between ibandronate (5.2%) and placebo (4.7%), and there were no reports of serious AEs (renal failure) in the active treatment group.

During the course of the study, 20% of patients (*n*=57) in the ibandronate 50 mg group and 15% of patients (*n*=42) in the placebo group died as a result of an AE. Death was most commonly due to malignancy progression, and no deaths were considered to be related to study treatment.

## DISCUSSION

The primary efficacy measure used in these two phase III trials of oral ibandronate was the SMPR, defined as the number of 12-week periods with new bone events, weighted for observation time. By assessing 12-week time periods where all complications are considered as a single occurrence, the SMPR avoids multiple counting of events, and therefore represents a conservative measure of efficacy. Clinical trials of other bisphosphonates in patients with metastatic bone disease have used the SRE or skeletal morbidity rate to assess the impact of treatment on skeletal complications. By counting all occurrences of new bone events, these measures may overestimate the effect of treatment, as many skeletal events (e.g. radiotherapy, fracture and bone surgery) are likely to be related in many cases.

The pooled results of the two oral trials demonstrated that ibandronate 50 mg once daily effectively reduces the incidence of new bone events in women with breast cancer and bone metastases. A statistically significant clinical benefit was observed for overall SMPR compared with placebo. This effect was maintained when skeletal events occurring in the first 12-week treatment period (including prescheduled radiotherapy events, which may have reduced the observed effect of active treatment) was included in the analysis. Ibandronate also significantly improved the need for bone radiotherapy and the need for bone surgery, both of which are considered to be highly clinically relevant indicators of disease outcomes. As the study was not powered to detect statistical significance on individual components of the SMPR, these results strongly support the clinical impact of ibandronate on the occurrence of new bone events. The results for fractures did not reach statistical significance in contrast to the data for i.v. ibandronate (Body *et al*, 2003a), and the patients had overall less fractures in the oral studies. Since a large meta-analysis of clinical trials has shown that bisphosphonates significantly decrease skeletal morbidity including fractures and need for radiotherapy (Ross *et al*, 2003), the trend that was observed for oral ibandronate will need to be confirmed in larger studies.

The percentage reduction in SMPR with oral ibandronate *vs* placebo in this pooled analysis (19%) was comparable to that observed in a clinical trial of i.v. ibandronate 6 mg (20%), which had a similar study design and was also conducted in patients with metastatic breast cancer (Body *et al*, 2003a, 2003c). The Poisson's regression analysis conducted on the pooled data set and the results of the i.v. trial also revealed comparable reductions in the risk of SREs with oral and i.v. ibandronate compared with placebo (hazard ratio 0.62, *P*=0.001 and hazard ratio 0.60, *P*=0.0033, respectively) (Body *et al*, 2003a, 2003c). Comparisons between these trials are cautious, as patients in the i.v. study had received a diagnosis of metastatic bone disease approximately 10 months earlier prior to study entry than patients in the oral trials, indicating that they had more severe disease. However, patients in the trials were similar in terms of their clinical presentation (age, baseline fracture incidence, performance status and bone-pain level), suggesting that the oral and i.v. formulations had broadly similar efficacy. Supporting this, oral and i.v. ibandronate were shown to have similar effects on secondary efficacy end points, with bone pain significantly reduced and maintained below baseline over 2 years of treatment, and significantly less deterioration in quality of life compared with placebo (Body *et al*, 2003b; Tripathy *et al*, 2003). This would be expected given that a daily oral 50 mg dose and an i.v. 6 mg given every 3–4 weeks provide the same bone surface exposure to ibandronate (Leyland-Jones, in press). Direct comparisons between bisphosphonates are difficult because of differences in study methodology. A comparative trial is currently examining the effects of oral ibandronate and i.v. zoledronate in patients with metastatic bone disease due to breast cancer.

Long-term drug safety and tolerability is an important consideration in the selection of treatment for skeletal metastases, due to the high disease-related morbidity burden and the side effects associated with systemic cancer therapy. Oral ibandronate 50 mg day^−1^ for 2 years of treatment was well tolerated in these trials, with an AE profile quite similar to placebo. As demonstrated for i.v. ibandronate (Body *et al*, 2003a; Lyubimova *et al*, 2003), oral ibandronate was not associated with renal AEs. This contrasts with the enhanced risk of renal AEs reported with i.v. zoledronate and pamidronate in a phase III trial (Rosen *et al*, 2001). With its benign renal safety profile, oral ibandronate may be used in patients with existing renal impairment. In addition, the results suggest that serum creatinine monitoring can be made, depending on the assessment of the individual patient, at the clinician's discretion. The associated reductions in renal monitoring time and costs could help to relieve the burden of bisphosphonate care on nursing staff and hospital budgets (Body, 2003).

As well as efficacy and safety, the availability of oral ibandronate could offer improved treatment flexibility for physicians and convenience for patients. Oral ibandronate may be prescribed alongside other oral agents (particularly hormonal treatment) for at-home dosing (e.g. when hospital care is not being received). Patients would no longer have to spend time travelling to and from the hospital solely for bisphosphonate infusion, allowing them to maintain their lifestyle without unnecessary disruption. The dosing regimen of oral ibandronate is convenient for patients. Adequate adherence is important in real-life situations, where dosing instructions are not closely monitored, unlike in clinical trials.

In conclusion, oral ibandronate 50 mg is an effective, well-tolerated and convenient treatment for the skeletal complications of metastatic bone disease.
